# Uncooled Short-Wave Infrared Sensor Based on PbS Quantum Dots Using ZnO NPs

**DOI:** 10.3390/nano9070926

**Published:** 2019-06-27

**Authors:** JinBeom Kwon, SaeWan Kim, JaeSung Lee, CheolEon Park, OkSik Kim, Binrui Xu, JinHyuk Bae, ShinWon Kang

**Affiliations:** 1 School of Electronics Engineering, College of IT Engineering, Kyungpook National University, 1370 Sankyuk-dong, Daegu 702-701, Korea; 2 Sensor System Research Center, Korea Institute of Science and Technology (KIST), 5 Hwarang-ro 14-gil, Seongbuk-gu, Seoul 02792, Korea

**Keywords:** Infrared, SWIR sensor, PbS, Quantum dots, Lead sulfide

## Abstract

Shortwave infrared (SWIR) sensors have attracted interest due to their usefulness in applications like military and medical equipment. SWIR sensors based on various materials are currently being studied. However, most SWIR detectors need additional optical filters and cooling systems to detect specific wavelengths. In order to overcome these limitations, we proposed a solution processed SWIR sensor that can operate at room temperature using lead chloride (PbS) QDs as a photoactive layer. Additionally, we adapted zinc oxide (ZnO) nanoparticles (NPs) as an electron transport layer (ETL) to improve the sensitivity of a PbS SWIR sensor. In this study, PbS SWIR sensors with and without a ZnO NPs layer were fabricated and their current–voltage (I–V) characteristics were measured. The on/off ratio of the PbS SWIR sensor with ZnO NPs was 2.87 times higher than that of the PbS SWIR sensor without ZnO NPs at the maximum current difference. The PbS SWIR sensor with ZnO NPs showed more stable current characteristics than that without ZnO NPs because of the ZnO NPs’ high electron mobility and proper lowest unoccupied molecular orbital (LUMO) level.

## 1. Introduction

Shortwave infrared (SWIR) sensors are currently used in various applications, including environmental monitoring, military equipment, and medical devices. Previously reported infrared sensors can be divided into photon detectors and thermal detectors based on their operating principles. Photon detectors based on materials such as HgCdTe and InSb (that convert electrons generated by light into signals) are fast and sensitive, but because their operating temperature is low, a separate cooling device is required and the equipment is quite expensive [1,2,3]. Thermal detectors based on materials such as VOx, amorphous silicon (a-Si) and Ti can operate at room temperature, but they need additional processes that convert temperature changes from infrared rays into signals and their sensitivity is low [4,5]. In addition, SWIR optoelectronics technology has been used as the structure for quantum well infrared photodetectors (QWIPs) because of the limitations imposed on integrated devices by the complicated epitaxial growth process of forming quantum dots (QDs) as well as the requirement for cooling devices and additional optic devices for stable operation [6,7,8,9]. To overcome the limitations of these types of sensors, many researchers have studied QDs based SWIR sensors that can be easy fabricated by the solution process [10,11]. QDs are applied to not only light emitting diode (LED) and solar cell but also various sensors such as gas and bio sensors because of their many advantages [12,13,14,15]. QDs can easily be made to adjust their targeted wavelength bands by controlling QDs core size due to the quantum confinement effect [16]. Moreover, QDs can be easily applied in solution processes such as ink-jet printing, contact printing, and spin-casting process, which are low cost, large-area process and can apply to flexible devices [17,18,19]. Among them, PbS QDs, which have absorbance in the infrared region and for which it is easy to select the absorption wavelength range through size control during synthesis, have been used as photoactive layers [20,21]. Zinc oxide (ZnO) nanoparticles (NPs) were used to reduce the high band gap difference between the PbS QDs and the aluminum (Al) electrode, thereby helping to emit electron-hole pairs formed by infrared rays and to improve the sensitivity of the sensor. ZnO NPs, which are used as an electron transport layer (ETL) in many fields such as LED devices and solar cells, can be easily synthesized at room temperature with facile particle size tuning and effectively transfer the electrons formed in the photoactive layer because of its high electron mobility and appropriate lowest unoccupied molecular orbital (LUMO) level (−4.2 eV) [22,23,24]. In addition, when a thin film is formed, it has high transparency and can thereby minimize the loss of light entering the device when the photodetector is fabricated. In this study, we proposed a solution processed SWIR sensor that can operate at room temperature using PbS QDs as a photoactive layer. To improve the sensitivity of the SWIR sensor, we adapted the ZnO NPs as an ETL by decreasing the bandgap difference between the PbS QDs and the Al electrode and improving the electron mobility. It is possible to process at room temperature, and it can operate at room temperature without a cooling device, which is a problem of the existing IR sensors. Thus, the optimized PbS SWIR sensor with ZnO showed an on/off ratio of 3.239 at the maximum current change, which is 2.87 times better than the SWIR sensor using only PbS QDs. 

## 2. Materials and Methods

### 2.1. Synthesis of Colloidal PbS QDs

The wavelength band of QDs can be tuned by controlling the size of their NPs. This takes advantage of the quantum confinement effect, a physical phenomenon in which the bandgap changes as a function of the NP size. In this study, we synthesized PbS QDs, having a wavelength band of 1330 nm, by using a colloidal method [25,26,27,28,29,30]. “Colloid” refers to a dispersion of particles in a gas or liquid that are larger than molecules or ions and they have at least one dimension between approximately 1 nm and 1 µm. To begin the PbS QDs synthesis, a mixture of 0.36 mmol of sulfur (S, 99.998%, Sigma-Aldrich, St. Louis, MO, USA) and 0.24 mL of oleylamine (OLA, 70%, Sigma-Aldrich, St. Louis, MO, USA) was stirred at room temperature for 30 min. After that, a mixture of 3.6 mmol of lead chloride (PbCl_2_, 99.999%, Sigma-Aldrich, St. Louis, MO, USA) and 2.4 mL of OLA was stirred in a 3-neck flask at room temperature under a flow of N_2_ gas (99.999%, Daeyang Gas, Inc., Busan, Korea) for 30 min. And the PbCl_2_–OLA mixture was heated to 160 °C for 1 h and it was cooled to 120 °C under vacuum for 20 min. Then, the prepared S–OLA solution and 225 μL of trioctylphosphine (TOP, 97%, Sigma-Aldrich, St. Louis, MO, USA) was quickly injected into the 3-neck flask under N_2_ gas flow. After allowing the chemical reaction to proceed at 100 °C for 1–360 min, the 3-neck flask cooled to room temperature. To remove the excess reagent that had not been incorporated during the synthesis, a mixture of the synthesized PbS QDs, 20 mL of butanol (99%, Sigma-Aldrich, St. Louis, MO, USA) and 10 mL of methanol (99.9%, Duksan Pharmaceutical CO. Ltd., Seoul, Korea) was centrifuged (FLETA-5, Hanil Scientific, Inc., Gimpo, Korea) at 3000 rpm for 10 min. Finally, the purified PbS QDs were dispersed in toluene (99.8%, Sigma-Aldrich, St. Louis, MO, USA) at 30 mg/mL.

### 2.2. Synthesis of ZnO NPs

The ZnO NPs used in this study were synthesized using an optimized sol-gel method. The sol-gel method is proven to have the advantages of reliability, repeatability, and ease of handling [31]. To proceed with the synthesis, 2.46 g of zinc acetate dehydrate (Zn(AC)_2_·2H_2_O, 98%, Sigma-Aldrich, St. Louis, MO, USA), as a Zn^2+^ precursor, was dissolved in 110 mL of methanol at 60 ℃. Then, 0.96 g of potassium hydroxide (KOH, 90%, Sigma-Aldrich, St. Louis, MO, USA) in 50 mL of methanol was gradually injected into the methanol solution (1 mL/s). After 1 h, the mixture became turbid, and the growth of ZnO NPs could be seen. To obtain uniform ZnO NPs, aging and rinsing process was performed by reacting with 160 mL of 2-propanol (99.9%, Duksan Pharmaceutical CO., Ltd., Seoul, Korea) and 800 mL of hexane (95%, Duksan Pharmaceutical CO., Ltd., Seoul, Korea) overnight [32,33]. The synthesized ZnO NPs were washed by centrifugation for 10 min at 3000 rpm to remove residues, such as K^+^. Finally, the synthesized ZnO NPs were dispersed in ethanol (99.9%, Samchun Pure Chemical CO., Ltd., Seoul, Korea) at 40 mg/mL, forming a transparent solution.

### 2.3. Device Fabrication

**Figure 1:**
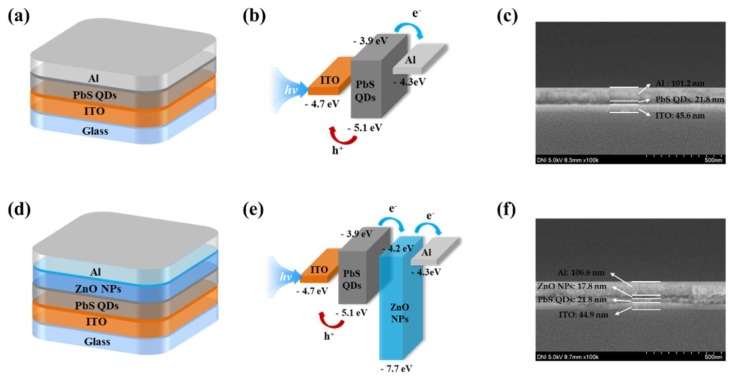
(**a**) Schematic device structure, (**b**) energy band diagram and (**c**) FE-SEM image of the PbS SWIR sensor without ZnO NPs, (**d**) schematic representation of the device structure, (**e**) energy band diagram and (**f**) FE-SEM image of the PbS SWIR sensor with ZnO NPs.

The PbS SWIR sensors were fabricated by spin-coating (LT-MS 200, LTS, Gyeonggi-do, Korea) method on glass substrates coated with a patterned indium tin oxide (ITO) anode. The ITO anode had a thickness of approximately 400 Å and a surface resistance of less than 12 Ω. Initially, to remove contamination on the ITO-patterned glass, the glass was cleaned with acetone, methanol, and deionized water and then exposed to UV ozone (AH-1700, AHTECH LTS Co., Ltd., Gyeonggi-do, Korea) for 15 min. To form the photo active layer, the PbS QDs solution was coated on the substrate, and annealed for 30 min at 110 °C in vacuum oven (ov–11, JEIO Tech, Daejeon Korea). To fabricate the PbS SWIR sensor with ZnO NPs, the ETL was formed by spin-coating ZnO NPs solution and annealed at 90 °C in a vacuum oven for 30 min (when fabricating the sensor without ZnO NPs, this process was omitted). The ETL can effectively transfer the electrons formed in the photoactive layer because of its high electron mobility and appropriate LUMO level of −4.2 eV. Finally, an aluminum (Al) cathode was deposited via thermal evaporation (OLED system, ULTECH, Daegu Korea) in a high-vacuum using a metal shadow mask. The Al electrode was more than 100 nm thick. The emissive area was 9 mm^2^, as defined by the cross section between the Al cathode and ITO anode. The current–voltage (I–V) characteristics of the PbS SWIR sensors were determined using a parameter analyzer (B1500A, Agilent, Santa Clara, CA, USA). Figure 1 shows the structure, energy band diagrams, and field emission scanning electron microscope (FE-SEM, SU8220, Hitachi, Japan) image of the fabricated sensors. It was confirmed that the thicknesses of the layers of the device without ZnO NPs were ITO: 45.6 nm, PbS QDs: 21.8 nm and Al: 101.2 nm, and those of that using ZnO NPs were ITO: 44.9 nm, PbS QDs: 21.8 nm, ZnO NPs: 17.8 nm and Al: 106.6 nm.

## 3. Results and Discussion

### 3.1. Characteristics of Synthesized PbS QDs

**Figure 2:**
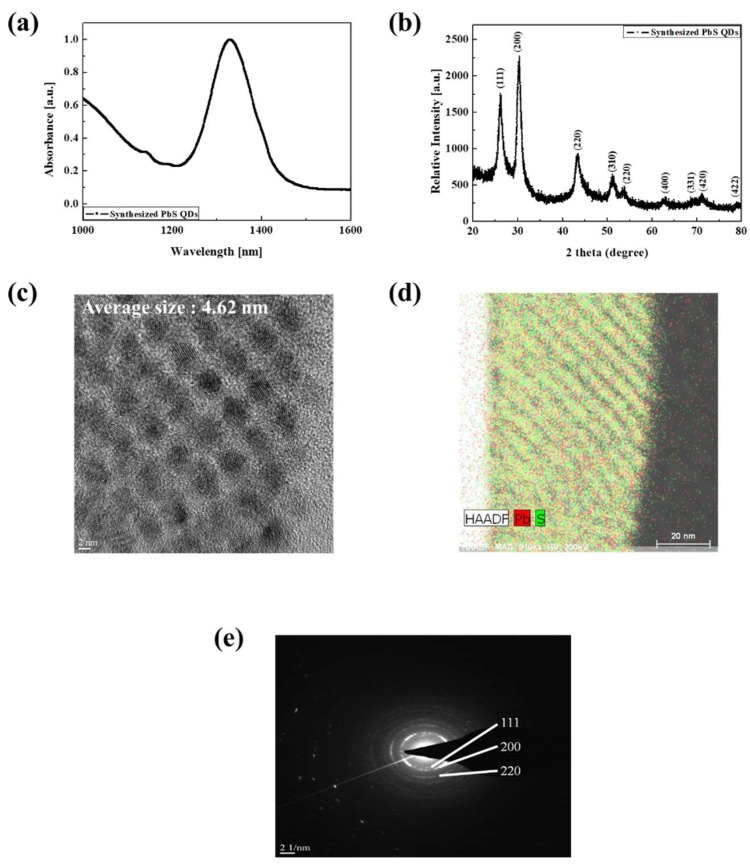
(**a**) Absorbance property, (**b**) XRD analysis, (**c**) TEM image, (**d**) composition analysis, and (**e**) diffraction ring of the synthesized PbS QDs.

As shown in Figure 2a, we measured the absorption spectra (Cary 5000 UV-Vis-NIR, Agilent, Santa Clara, CA, USA) of the synthesized PbS QDs and confirmed that they have an absorption peak at wavelength λ = 1330 nm. This result means that the synthesized PbS QDs can absorb 1330 nm wavelength light and generate electron-hole pairs (EHPs) when irradiated by an IR light source. To confirm that PbS QDs were indeed synthesized and to compare them with previously reported PbS QDs, X-ray diffraction (XRD, Max-2500, Rigaku, Japan) analysis was performed. The sample for XRD analysis of PbS QDs was formed by spin-coating process on glass substrate (10 mm × 10 mm). As shown in Figure 2b, the XRD result showed the same result as reported, confirming that the PbS QDs were well synthesized [34,35,36]. The following Equation (1) is the Scherrer equation used to calculate the nanocrystal size based on the XRD result and the synthesized PbS QDs calculated.

(1) $$D_{hkl}\;\left(nm\right)=\frac{K\cdot \lambda }{\beta \cos\theta }$$

where *K* is the shape constant, *λ* is the wavelength of the X-ray, *β* is the full width at half maximum (FWHM), and *θ* is the half value between the incident angle and the scattered X-ray wavelength vector [37,38,39]. We confirmed that their size calculated by Equation (1) was 4.85 nm.

To confirm the formation and composition of the QDs, field-emission transmission electron microscope (FE-TEM, Titan G2 ChemiSTEM Cs Probe, FEI Company, Hillsboro, OR, USA) images were examined. For TEM measurement, the sample was fabricated by spin coating PbS QDs solution on a 10 mm × 10 mm glass substrate and it was cut into 10 μm × 1 μm with width of 70 nm by focused ion beam (FIB, Versa3D LoVac, FEI Company, Hillsboro, OR, USA). From Figure 2c–e, we can see that the synthesized QDs were formed uniformly, and the composition analysis of the TEM image showed that the cores were composed of Pb and S. To determine the size of PbS QDs, we analyzed the TEM image by ImageJ (National Institutes of Health) program, which shows the area of particles in the photograph and calculated their diameter. In the results, 28 PbS QDs of 4 nm to 5 nm were identified and their average size was 4.62 nm, which is similar to the size calculated by the XRD result. The diffraction ring is consistent with the XRD measurement result and confirmed that the PbS QDs has a plane-centered cubic structure [40,41,42].

### 3.2. Characteristics of Synthesized ZnO NPs

**Figure 3:**
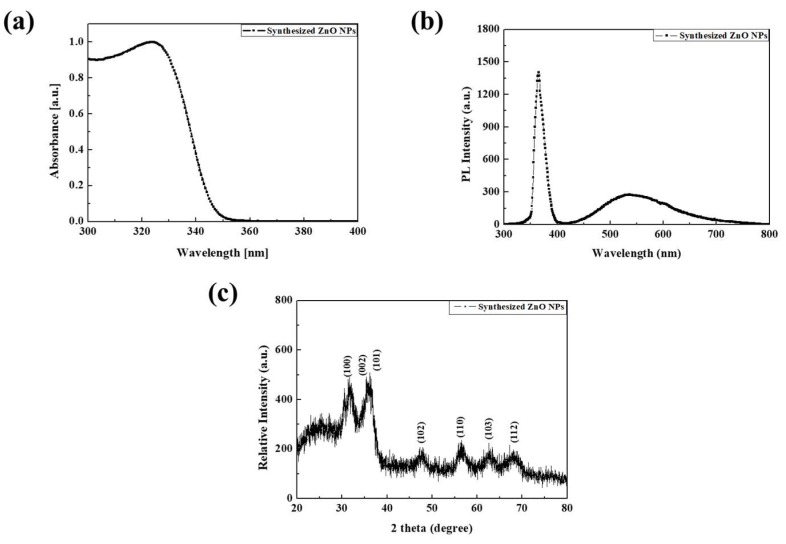
(**a**) Absorbance property, (**b**) PL characteristic and (**c**) XRD analysis of the synthesized ZnO NPs.

In order to confirm the size and characteristics of the synthesized ZnO NPs, we measured the absorption spectrum (Cary 5000 UV-Vis-NIR, Agilent, Santa Clara, CA, USA), the photoluminescence (PL) characteristics (QE 65000, Ocean optics Inc., Large, FL, USA), and XRD patterns. Figure 3a shows the UV-vis absorption spectra of the ZnO NPs. We confirmed that the synthesized ZnO NPs has a broad absorption band with absorption peak of 323.5 nm in the UV region. Figure 3b shows the PL characteristic of the ZnO NPs has two peaks at 365 nm and 500 nm. The UV radiation, at around 365 nm, originated from the direct bandgap of the ZnO NPs. The other peak, at around 500 nm, originated from the deep trap state on the surface of the ZnO NPs, including oxygen vacancies [43]. To verify the crystal structure, the XRD result of the synthesized ZnO NPs was measured. As shown in Figure 3c, the peak values of the ZnO NPs were measured at 31.7 (100), 34.4 (002), and 36.25 (101), indicating that the synthesized ZnO has a hexagonal wurtzite structure [44,45]. Using the XRD results, the size of the NPs was calculated by the Scherrer Equation (1) to be 4.79 nm.

### 3.3. Performance of the SWIR Sensors

**Figure 4:**
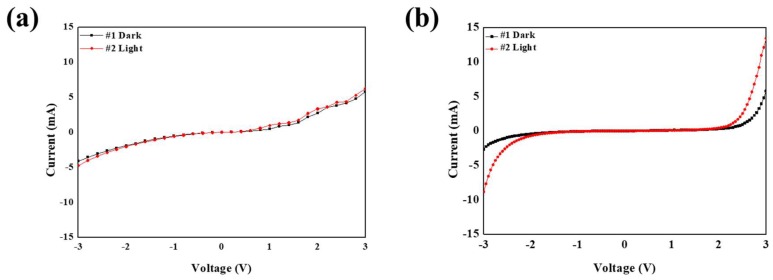
I–V characteristics of (**a**) PbS SWIR sensor without ZnO NPs, and (**b**) PbS SWIR sensor with ZnO NPs.

To measure the performance of the fabricated PbS SWIR sensors, the I–V characteristics were measured. When IR light irradiated the PbS SWIR sensor, the EHPs generated at the photo active layer were extracted to the electrodes by the external electric field. The dark current was measured when the IR light source (SL-5, StellarNet, Inc., Tampa, FL, USA) was turned off, and the light current was measured when the IR light source was turned on. The voltage was swept in the range of −3 V to 3 V. Figure 4a,b show the I–V characteristics of the fabricated PbS SWIR sensors. The on/off ratio of the PbS SWIR sensor without ZnO NPs was 1.147 at the maximum current difference, with a dark current of −4.1409 mA and a light current of −4.7533 mA. The on/off ratio of the PbS SWIR sensor with ZnO NPs was 3.293 at the maximum current difference, with a dark current of −2.6899 mA and a light current of −8.8582 mA. In addition, the PbS SWIR sensor with ZnO showed a more stable current characteristic than that without ZnO. As these results show, PbS QDs can detect IR light and ZnO NPs can improve the sensitivity and current stability of the PbS SWIR sensor because of the ZnO NPs’ high electron mobility and proper LUMO level. 

## 4. Conclusions

We synthesized PbS QDs and ZnO NPs to improve the sensitivity and current stability of SWIR sensors. Then, a solution-processed PbS SWIR sensor was fabricated with ZnO NPs and compared to a PbS SWIR sensor without ZnO NPs. The sensor with ZnO NPs had a more sensitive and stable I–V characteristic than the PbS SWIR sensor without ZnO NPs. From measuring the I–V characteristics according to voltage sweep from −3 to 3 V, the on/off ratio of the PbS SWIR sensor with/without ZnO NPs were 1.147, 3.293, respectively, at the maximum current difference. These results confirmed that the on/off ratio of a PbS QDs SWIR sensor using ZnO NPs is 2.871 times higher than that of a PbS SWIR sensor without ZnO NPs. Moreover, the PbS SWIR sensor with ZnO NPs presented a more stable current characteristic than the PbS SWIR sensor without ZnO NPs.
